# The effect of selenium supplementation on oxidative stress, clinical symptoms and mental health status in patients with migraine: a study protocol for a double-blinded randomized clinical trial

**DOI:** 10.1186/s13063-024-08018-8

**Published:** 2024-03-22

**Authors:** Arghavan Balali, Omid Sadeghi, Javad Anjom-Shoae, Mohammad Hossein Rouhani, Fariborz Khorvash, Gholamreza Askari

**Affiliations:** 1 Student Research Committee, Isfahan University of Medical Sciences, Isfahan, Iran; 2 Department of Community Nutrition, School of Nutrition and Food Science, Nutrition and Food Security Research Center, Isfahan University of Medical Sciences, Isfahan, Iran; 3 Adelaide Medical School, Faculty of Health and Medical Sciences, Adelaide, Australia; 4 Neurology Research Center, School of Medicine, Isfahan University of Medical Sciences, Isfahan, Iran

**Keywords:** Antioxidant, Migraine, Oxidative stress, Randomized controlled trial, Selenium

## Abstract

**Background:**

Despite a number of recommended strategies, effective treatment of migraine remains elusive. Given the role of oxidative stress in the pathogenesis of migraine, selenium, as an antioxidant nutrient, may have a beneficial effect on migraine outcomes. However, no study has explored the effects of selenium supplementation on migraine symptoms, oxidative stress biomarkers, and mental health. Therefore, this randomized, double-blinded, placebo-controlled clinical trial aims to examine the effects of selenium supplementation among migraine patients.

**Methods:**

**S**eventy-two migraine patients will receive either 200 µg/day selenium supplement (*n* = 36) or placebo (*n* = 36) for 12 weeks in a randomized, double-blinded, placebo-controlled study. The severity, frequency, and duration of headaches, mental health indices including depression, anxiety, and distress, and quality of life, as well as biomarkers of oxidative stress such as nitric oxide (NO), malondialdehyde (MDA), total antioxidant capacity (TAC), and total oxidant status (TOS), will be measured at the baseline and end of the study. The intention-to-treat (ITT) approach will be used to estimate missing values. One-way analysis of covariance (ANCOVA) will be performed to detect the effect of selenium supplementation on outcome variables.

**Discussion:**

Oxidative stress is recognized as a key contributor to migraine pathogenesis. Selenium is an essential trace element with antioxidant properties, capable of crossing the blood–brain barrier (BBB), holding promise to alleviate the oxidative stress and neurotoxicity. Thus, selenium may beneficially affect clinical symptoms and oxidative stress as well as the quality of life in migraine patients.

**Trial registration:**

This trial was registered in the Iranian Registry of Clinical Trials (https://www.irct.ir/) on 27 May 2023 with the code number IRCT20121216011763N60.

**Supplementary Information:**

The online version contains supplementary material available at 10.1186/s13063-024-08018-8.

## Introduction

### Background and rationale {6a}

Migraine is a prevalent neurological disorder affecting approximately 14–15% of the global population [1], which imposes a substantial burden on public health. In Iran, the prevalence of migraine is estimated to be 15.1% among adults [2]. Migraine is also known as the second leading cause of disability in individuals below 50 years, particularly among women, and is associated with an increased risk of different psychological disorders, including depression and anxiety [3, 4]. Migraine patients experience moderate to severe, throbbing and unilateral headaches, which are frequently accompanied by other symptoms, such as vomiting, nausea, photophobia, and phonophobia [5]. Accordingly, finding effective strategies for the management of migraine is urgently required.

Despite the availability of various pharmacotherapies for migraine management, including beta-blockers, calcium channel antagonists, triptans, antipsychotics, antiepileptics, and botulinum neurotoxins [6], their efficacy is often limited, and accompanied by several side effects [7]. Therefore, complementary therapies such as vitamin/mineral supplementation, acupuncture, and herbal medicine have received more attention. Recent evidence indicates that oxidative stress and inflammation play a key role in the pathophysiology of migraine headaches by stimulating abnormal activation of nociceptive neurons. In addition, migraine headaches are recognized as a neuro-protective response to elevated oxidative stress and inflammation in the brain [8]. Previous studies have shown that antioxidants such as vitamin C, zinc, and coenzyme Q10 benefit oxidative stress and clinical symptoms of migraine patients [9].

Of different antioxidants, selenium is of particular interest, exerting its biological functions through selenoproteins such as glutathione peroxidase, iodothyronine deiodinases, and thioredoxin reductase [10, 11]. A number of studies have shown that selenium plays a key role in the normal physiological function of the brain [12]. Furthermore, the established antioxidant activity of selenoproteins in the central nervous system suggests that lower selenium levels can be associated with brain damage [13]. Indeed, a recent case–control study showed that migraine patients have lower selenium levels than controls, reporting that subjects in the lowest quartile of selenium are eleven times more likely to have migraine compared with those in the top quartile [14]. Given the role of oxidative stress in the pathogenesis of migraine [15, 16], selenium may have a beneficial effect on migraine outcomes. However, despite evidence on the beneficial effects of selenium supplementation on other neurodegenerative diseases such as cognitive disorders [17], there has been no study assessing the effect of selenium supplementation on migraineurs.

### Objectives {7}

The present study aims to investigate the effect of selenium supplementation on oxidative stress, mental health and clinical symptoms in migraine patients.

### Trial design {8}

A randomized, parallel, two-arm, double-blind, and placebo-controlled superiority clinical trial will be conducted.

## Methods: Participants, interventions, and outcomes

### Study setting {9}

The present study will be conducted at the neurology clinic of Al-Zahra Hospital, affiliated with Isfahan University of Medical Sciences, Isfahan, Iran.

### Eligibility criteria {10}

#### Inclusion criteria

Participants who (1) have a confirmed migraine diagnosis according to the International Classification for Headache Disorders (ICHD-3) criteria [18] by an expert neurologist, (2) are aged between 18 and 65 years, and (3) experience at least two migraine attacks per month, will be included. No restrictions on the type of migraine will be considered; thus, patients with all types of migraine headaches, including migraine with/without aura and episodic/chronic migraine headaches, will be included.

#### Non-inclusion criteria

Participants who (1) are pregnant or lactating; (2) have consumed antioxidant supplements in the last three months; (3) have a medical history of infectious disorders, autoimmune disorders, diabetes mellitus, cardiovascular diseases, or any other neurological disorders such as other subtypes of headaches (i.e. tension-type headache, etc.), epilepsy, Parkinson’s and multiple sclerosis, or (4) are current smokers, will not be included.

#### Exclusion criteria

Participants who (1) suffer from complications related to selenium supplementation, (2) change the type or dosage of their medications during the intervention, and (3) are not willing to continue the intervention, will be excluded.

### Who will take informed consent? {26a}

Each participant will be informed about the purpose, benefits and risks of the investigation by the principal investigator (AB) to obtain written informed before participation.

### Additional consent provisions for collection and use of participant data and biological specimens {26b}

Participants will be requested to provide consent for the review of their medical records and blood sample collection to assess oxidative stress biomarkers, including nitric oxide (NO), total antioxidant capacity (TAC), total oxidant status (TOS), and malondialdehyde (MDA). Participants will also provide consent for using their data in future publications, which is not obligatory.

## Interventions

### Explanation for the choice of comparators {6b}

Participants will be randomly assigned to either the intervention or placebo group. The intervention group (*n* = 36) will receive a daily tablet containing 200 µg selenomethionine for 12 weeks. In parallel, the placebo group (*n* = 36) will receive a tablet containing 200 µg starch per day for 12 weeks.

### Intervention description {11a}

After the recruitment of participants, they will be randomly assigned to either group. Participants in the intervention group will receive a tablet containing 200 µg selenomethionine daily for 12 weeks. Those in the placebo group will receive a tablet containing 200 µg starch per day. Starch was chosen given its easily digestible, and has a similar appearance and taste to selenium. The selenium and placebo tablets will look identical, having the same shape, color, and size. To maintain the study's integrity, an unbiased person will label the packs as A or B without knowing the contents. Participants will be asked not to change their lifestyle, including physical activity, dietary habits, and medication during the study. Clear instructions will also be provided on how to use their supplements. To monitor progress and address any concerns, participants will have phone check-ins every two weeks to track supplement consumption and assess for potential side effects.

### Criteria for discontinuing or modifying allocated interventions {11b}

Participants will be free to withdraw from the study at any time. In addition, subjects may be withdrawn at the discretion of the investigator if the subject is non-compliant with the protocol. Further detailed information will be explained in the study results.

### Strategies to improve adherence to interventions {11c}

Participants will be provided with selenium and placebo tablets during weeks 1 and 6. At baseline, instructions on how to use their supplements will be provided. They will be asked to return the remaining tablets at week six and end of the trial. Moreover, any side effects that they experience will be recorded. Adherence to intervention refers to how participants’ behavior aligns with their assigned intervention, which will be evaluated by phone call and counting the returned tablets at the end of the trial. Compliance will be calculated using the following formula:

$$\mathrm{Compliance rate}=\left(\mathrm{tablets taken}/\mathrm{tablets prescribed}\right)\times 100$$

### Relevant concomitant care permitted or prohibited during the trial {11d}

The routine care and treatment provided by a neurologist, will remain unchanged during the study. Participants will only receive selenium supplements or a placebo in addition to standard treatments.

### Provisions for post-trial care {30}

Selenium or placebo supplements will only be administered for 12 weeks. Regular care and treatments will also continue after the trial concludes.

### Outcomes {12}

The primary outcome of the present study is to investigate the effect of selenium on oxidative stress and clinical symptoms in migraine patients. Secondary outcomes include mental health status and participants’ quality of life.

### Participant timeline {13}

**Fig. 1:**
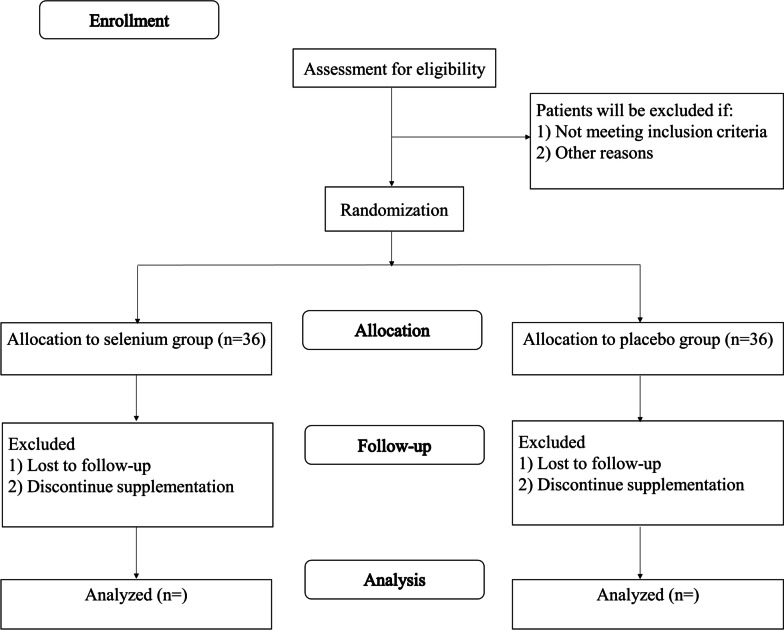
The protocol flowchart and timeline of the study

**Fig. 2:**
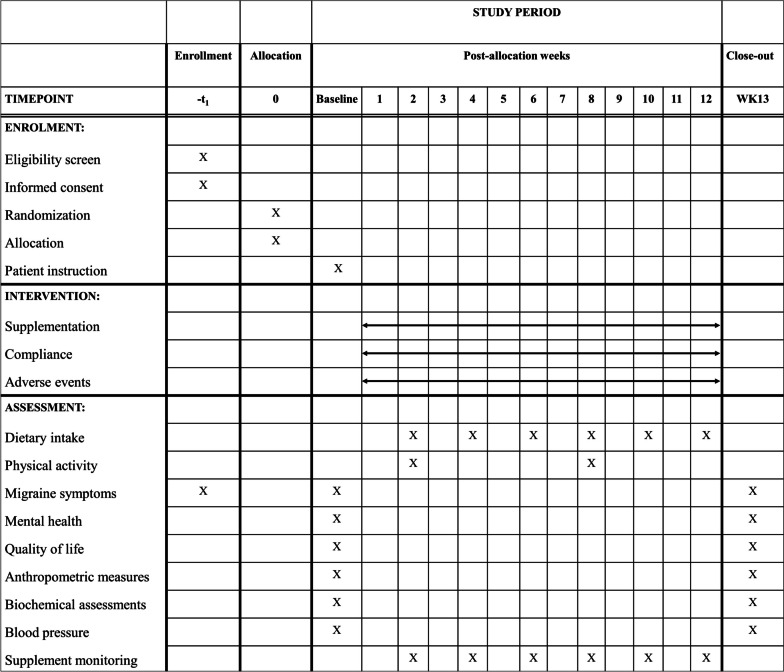
Schedule of enrolment, intervention, and assessment based on SPIRIT guidelines. The “X” refers to what is done in the given period. Abbreviations: WK, week

The study flow chart and timeline are presented in Figs. 1 and  2, respectively. The study protocol was designed and developed in accordance to the Standard Protocol Items: Recommendations for Interventional Trials (SPIRIT).

### Sample size {14}

The sample size was calculated based on means and standard deviations for the primary variable, which is MDA levels, reported in the study of Razavi et al. [19], considering a type I error of 5% (α = 0.05) and type II error of 20% (β = 0.20, power = 80%). Sample size was calculated using the following formula [20]:

$$n=\frac{2{(\mathrm{a }+\mathrm{ b})}^{2} {\upsigma }^{2}}{{(\upmu 1 -\upmu 2)}^{2} }=33$$

*n* = the sample size in each group.

μ1 = the mean for malondialdehyde in the intervention group: -0.13 μmol/L based on the study of Razavi et al. [19]).

μ2 = the mean for malondialdehyde in the control group: 1.4 μmol/L based on the study of Razavi et al. [19]).

σ = the variance (SD) for mean concentrations of malondialdehyde: 4.00 (the average SDs reported for malondialdehyde in the control and intervention groups of Razavi et al.’s study [19]).

a = the conventional multiplier for alpha = 0.05 that was 1.96.

b = the conventional multiplier for power = 0.80 that was 0.842.

Based on the formula, we will need a sample size of 33 for each group. Considering a drop-out rate of 10%, we plan to recruit 36 migraine patients in each group.

### Recruitment {15}

Participants will be recruited from individuals who were referred to the neurology clinic of Al-Zahra Hospital, affiliated with Isfahan University of Medical Sciences, Isfahan, Iran. An expert neurologist will evaluate the diagnosis of migraine according to ICHD-3 criteria, and those with a definite migraine diagnosis will be then screened according to the inclusion/exclusion criteria by the principal investigator (AB). All eligible participants will be asked to provide written informed consent.

## Assignment of interventions: Allocation

### Sequence generation {16a}

After recruitment, stratified block randomization will be performed based on participants’ age (18 to 39 and 40 to 65 years) and sex (male or female) across the two groups. Then, within each of the aforementioned strata, eligible participants will be randomly assigned in a 1:1 ratio to receive either selenium or placebo. Balanced randomization will be generated using an online tool (https://www.sealedenvelope.com/simple) by a person who is not involved in the study. Participants in the intervention group (*n* = 36) will receive a tablet containing 200 µg selenomethionine daily, while the placebo group (*n* = 36) will receive a tablet containing 200 µg starch daily.

### Concealment mechanism {16b}

An independent person who is not involved in the trial will label supplement packs as A and B. Both the investigators who are performing the study and participants will be blinded to the intervention allocation. Additionally, the allocation sequence will be concealed from those who are assigning participants to the intervention groups using opaque sealed envelopes. Randomization codes will be unlocked after the trial completion.

### Implementation {16c}

Participants will be allocated to either the selenium or placebo group by an independent investigator who is not involved in the study. The allocation process will be monitored by a second researcher (OS).

## Assignment of interventions: Blinding

### Who will be blinded {17a}

The current study will have a double-blind design, which means that both the investigators who are performing the study and participants will be unaware of group allocation.

### Procedure for unblinding if needed {17b}

Labels on supplement packs will be revealed only after the study completion. However, based on the criteria of the Medical Ethics Committee, unblinding is allowed in case of any adverse events to determine whether the patient received selenium or placebo. If so, it will be recorded and reported in the Iranian Registry of Clinical Trials, and the Ethics Committee of Isfahan University of Medical Sciences, as well as results of the study. Further information will also be collected to decide whether those who reported adverse side effects should be excluded.

## Data collection and management

### Plans for assessment and collection of outcomes {18a}

#### Demographic variables

A general self-administered questionnaire will be used to assess participants’ demographic characteristics, including age, sex, marital status, education, medical history and their medications, family history of migraine, supplementation, and smoking at the beginning of the study. Moreover, the socioeconomic status of subjects will be evaluated through specifically designed questions such as the number of family members, home or car ownership, and participant’s job using a self-administered questionnaire.

#### Migraine symptoms assessment

An expert neurologist will assess migraine characteristics, including severity, frequency, and duration of migraine attacks. Headache severity will be assessed using a visual analogue scale (VAS) questionnaire on a 0–10 scale with 0 representing "no pain" and 10 "the worst imaginable pain”. The Persian version of this questionnaire, validated by previous investigators, will be used [21]. The patients will note the feeling that represents their perception of pain. The frequency and duration of migraine headaches will be evaluated as the number of attacks per month and the mean duration of attack in each day, respectively.

#### Dietary intake assessment

Six dietary recalls (four weekdays and two weekend days) will be obtained from all participants at weeks 2, 4, 6, 8, 10, and 12. All participants will be asked to report their dietary intake based on household measures. Then, these reports will be converted to grams using standard protocols [22]. To calculate nutrient intake throughout the trial, we will use the United States Department of Agriculture (USDA) nutrient databank.

#### Physical activity assessment

In addition, two 1-day physical activity (PA) records (including a working day and a non-working day) will be collected from all participants at weeks 2 and 8. PA levels will be reported as metabolic equivalent hours per day (MET/h/day).

#### Anthropometric measures and blood pressure

Body weight will be measured to the closest 100 g using a digital scale at the state of minimum clothing and without shoes. Height will be assessed to the nearest 1 mm using a non-stretched tape in a standing position with no shoes. Waist circumference (WC) will be measured to the nearest 1 mm at the midpoint between the lowest rib and the iliac crest, with the subject standing, after a normal exhalation. Body mass index (BMI) will be calculated as weight in kilograms divided by height squared in meters. Blood pressure (BP), including systolic and diastolic BP, will be assessed using a standard sphygmomanometer. Measurements will be taken twice, and the average value will be reported as the final BP.

#### Assessment of quality of life

There is some evidence indicating that quality of life is impaired in migraine patients [23]. In the present study, we will use two widely used questionnaires, including the Headache Impact Test (HIT-6) and the Migraine-specific Quality of Life (MSQ), to investigate the effect of selenium supplementation on the participants’ quality of life. As different aspects of functioning are examined in each questionnaire, using these two concurrently is common in clinical trials [24, 25]. These questionnaires have been translated into Persian by prior investigators, with their validity and reliability confirmed among Iranian adults [26, 27].

The HIT-6 questionnaire evaluates the influence of headaches on migraine patients’ well-being and daily performance [28]. This questionnaire includes six items, each with five response options, scored between 6 and 10, including never (six points), rarely (eight points), sometimes (10 points), very often (11 points), and always (13 points) options. The total score of HIT-6 ranges from 36 to 78. It is categorized as no impact (36–49), moderate impact (50–55), substantial impact (56–59), and severe impact (≥ 60) on patients' quality of life.

The MSQ (version 2.1) explores the impact of migraine headaches on patients’ daily functioning over the past four weeks [29]. This questionnaire has three domains: 1) role restrictive domain describing the reduction of daily activities (7 items), 2) role preventive domain describing the influence of migraine headaches on normal work and social activities (4 items), and 3) emotional functioning domain that evaluates the emotions associated with headache (3 items). Each item includes six response options, scored between 1 and 6: none of the time (score 1), a little bit of the time (score 2), some of the time (score 3), a good bit of the time (score 4), most of the time (score 5), and all of the time (score 6).The total score of each domain is computed as a sum of item responses and rescaled from 0 to 100, where higher scores indicate better quality of life.

#### Mental health assessment

To assess psychological disorders, a 21-item Depression, Anxiety, and Stress Scale (DASS-21) will be used [30]. The validity and reliability of the Persian version of this questionnaire have been also approved by Samani et al. [31]. Each subscale (depression, anxiety, and distress) consists of seven questions, with responses rated from 0 (does not apply to me at all) to 3 (applies to me most of the time). Subscale scores range from 0 to 21, with higher scores indicating increased psychological distress. To align with DASS-42, subscale scores will be multiplied by two for interpretation.

### Plans to promote participant retention and complete follow-up {18b}

To enhance participant retention, the potential health benefits of selenium will be discussed, and regular telephone contact by the principal investigator (AB) will be maintained.

### Data management {19}

Data coding, security, and storage will be monitored by one of the researchers. Electronic data entry will occur at the participating site, with secure storage of forms. Data entry and values will undergo double-checking.

### Confidentiality {27}

Each participant will receive a unique identification code to store research data. The code list will be accessible only to researchers and protected by the principal investigator throughout the trial. Participants’ personal details will remain confidential in all publications.

### Plans for collection, laboratory evaluation, and storage of biological specimens for genetic or molecular analysis in this trial/future use {33}

A 5 ml blood sample will be collected at the baseline and end of the intervention. The serum will be obtained by centrifugation immediately after sample collection, then will be maintained at -80 degrees Celsius for future tests. NO levels will be measured by the Griess method, serum levels of TAC by the CUPric Reducing Antioxidant Capacity, TOS by the modified Ferrous Oxidation-xylenol Orange, and MDA levels by the Thiobarbituric acid reactive substance using commercial kits (Kiazist Life Sciences, Iran).

## Statistical methods

### Statistical methods for primary and secondary outcomes {20a}

To examine normal distribution of variables, the Kolmogorov–Smirnov test will be used. To normalize the non-normally distributed variables, we will conduct a log transformation. The intention-to-treat (ITT) approach will be used to estimate missing values on outcome variables. Independent sample t-test will be used to detect differences in quantitative variables between intervention and control groups. For the assessment of qualitative variables between the two groups, the chi-square test will be performed. The paired-sample t-test will also be used to examine within-group differences. One-way analysis of covariance (ANCOVA) will be performed to detect the effect of selenium supplementation on outcome variables. In this analysis, potential confounders will be taken into account. All statistical analyses will be conducted using the SPSS software version 21 (SPSS, Inc. Chicago, IL, USA). P-values less than 0.05 will be considered statistically significant.

### Interim analyses {21b}

No interim analyses are planned for the present study; however, immediate study cessation will occur for those with adverse side effects. These adverse side effects include death, disability, and severe allergic reactions, which have a significant impact on the patient's health. However, as the ITT analysis will be performed, participants who drop out will also be included in data analysis.

### Methods for additional analyses (e.g., subgroup analyses) {20b}

No subgroup analyses are planned. ANCOVA will be performed to detect the effect of selenium supplementation on outcome variables, which will be adjusted for variables that significantly differ between the selenium and placebo groups.

### Methods in analysis to handle protocol non-adherence and any statistical methods to handle missing data {20c}

In the present study, participants who drop out will not be excluded from the final analysis as an ITT method will be conducted. Missing data will be addressed by multiple imputations.

### Plans to give access to the full protocol, participant-level data, and statistical code {31c}

The corresponding author will make datasets, statistical code, and the complete protocol available upon reasonable request.

## Oversight and monitoring

### Composition of the coordinating center and trial steering committee {5d}

The principal researcher coordinates the study, with regular communication among the study team and supervision by a steering committee. Furthermore, the Ethics Committee of Isfahan University of Medical Sciences will ensure adherence to ethical principles, and study will be modified or terminated in case of ethical violations.

### Composition of the data monitoring committee, its role, and reporting structure {21a}

Data monitoring will be conducted impartially and continuously by the academic committee of Isfahan University of Medical Sciences.

### Adverse event reporting and harms {22}

Based on previous studies, supplementation with 200 µg of selenium per day has been found to be safe. If any complications were reported with the selenium supplementation, the intervention will be immediately stopped, and the necessary medical measures will be taken.

### Frequency and plans for auditing trial conduct {23}

The present study will be conducted by the guidance of the Ethics Committee of Isfahan University of Medical Sciences. The quality, validity, and adherence to ethical standards will be monitored by this Committee during at least two monitoring sessions. In addition, a report will be submitted to the auditor every three months.

### Plans for communicating important protocol amendments to relevant parties (e.g., trial participants, ethical committees) {25}

Any changes to the study protocol details, including study sample size or methods, must be approved by the Ethics Committee of Isfahan University of Medical Sciences. Also, all changes will be reported at https://www.irct.ir/.

### Dissemination plans {31a}

Final findings and data will be presented in future publications.

## Discussion

There has been no globally approved treatment for migraine until now, presenting a considerable challenge for healthcare professionals [32]. The management of migraine is particularly complex due to its high prevalence and associated comorbidities, especially mental disorders [4]. Therefore, the development of safe, non-pharmacological, nutrient-based approaches for the management of migraine headaches is critical. Considering the beneficial effects of selenium in different neurological disorders, particularly those with inflammatory and oxidative nature, supplementation with this nutrient could be advocated as the potential strategy for the management of migraine headaches [12]. To the best of our knowledge, this is the first clinical trial evaluating the effect of selenium supplementation on migraine symptoms, psychological disorders, and biomarkers of oxidative stress in migraine patients.

The underlying rationale is based on the hypothesis that selenium may positively affect migraine symptoms through scavenging free radical species, particularly NO, and elevating antioxidant levels in the brain. In the past few decades, nutraceutical agents such as antioxidants have attracted much attention for migraine management. A limited number of studies have investigated the effects of various antioxidants on migraine symptoms, mostly reporting promising findings. For example, in one of these trials, nano-curcumin and coenzyme Q10 supplementation were shown to reduce the frequency, duration, and severity of migraine headaches [33]. Similar effects on the frequency and severity of headaches were also observed after supplementation with a combination of different antioxidants, including pine bark extract, vitamin C, and vitamin E, among migraine patients [34]. In addition, a recent clinical trial, in which the effect of vitamin C, E, and N-acetylcysteine co-supplementation on migraine patients was assessed, a significant reduction in headache frequency and duration was also reported [35]. Taken together, there is a need for a clinical trial investigating the effect of selenium supplementation on migraine symptoms and other clinical parameters of migraine patients.

## Strengths

The current study is the first clinical trial evaluating the effect of selenium supplementation on migraine symptoms, mental disorders, and biomarkers of oxidative stress in a relatively large sample of migraine patients. In this study, several outcomes including clinical, anthropometric, and biochemical parameters will be assessed at the baseline and end of the study. In addition, based on previous studies, the intervention duration of our study will be sufficient to investigate the effects of selenium supplementation on migraine outcomes [34, 35].

## Limitations

Despite using validated questionnaires for the assessment of dietary intake, physical activity, migraine symptoms, mental health, and quality of life, the misclassification bias cannot be completely ruled out. Moreover, due to using a recall-based questionnaire for the assessment of dietary intakes, energy intake is likely to be underestimated. In addition, due to ethical issues, we will not be able to assess the effect of selenium supplementation as a monotherapy.

## Trial status

The recruitment phase of this study commenced on 30 May 2023, and the anticipated last patient/last visit date is 29 February 2024 (Protocol version number: 02, Date: 2024/02/17). Different challenges related to the rising costs of selenium supplements and laboratory assessments in Iran led to an earlier project start, thus, we could not submit the manuscript before recruitment.

### Supplementary Information

**Supplementary Material 1.**

## Data Availability

Any data required to support the protocol will be supplied on reasonable request by the corresponding author.

## Abbreviations

NO: Nitric oxide
MDA: Malondialdehyde
TAC: Total antioxidant capacity
TOS: Total oxidant status
ICHD-3: International Classification for Headache Disorders
USDA: United States Department of Agriculture
WC: Waist circumference
BMI: Body mass index
BP: Blood pressure
VAS: Visual analogue scale
MSQ: Migraine-specific quality of life
HIT: Headache impact test
DASS: Depression, anxiety, and stress scale
